# G protein βγ subunits play a critical role in the actions of amphetamine

**DOI:** 10.1038/s41398-019-0387-8

**Published:** 2019-02-11

**Authors:** J. C. Mauna, S. S. Harris, J. A. Pino, C. M. Edwards, M. R. DeChellis-Marks, C. D. Bassi, J. Garcia-Olivares, S. G. Amara, F. G. Guajardo, R. Sotomayor-Zarate, M. Terminel, E. Castañeda, M. Vergara, T. Baust, E. Thiels, G. E. Torres

**Affiliations:** 1 Department of Neurobiology, University of Pittsburgh School of Medicine, Pittsburgh, PA USA; 2 Department of Pharmacology and Therapeutics, University of Florida College of Medicine, Gainesville, FL USA; 3 Laboratory of Cellular and Molecular Neurobiology, National Institute of Mental Health, NIH, Bethesda, MD USA; 4 Laboratory of Neurochemistry and Neuropharmacology, Center for Neurobiology and Brain Plasticity, Universidad de Valparaíso, Valparaíso, Chile; 5 Department of Psychology, University of Texas at El Paso, El Paso, TX USA; 6 Center for Addiction Research and Education, University of Florida College of Medicine, Gainesville, FL USA

## Abstract

Abnormal levels of dopamine (DA) are thought to contribute to several neurological and psychiatric disorders including drug addiction. Extracellular DA levels are regulated primarily via reuptake by the DA transporter (DAT). Amphetamine, a potent psychostimulant, increases extracellular DA by inducing efflux through DAT. Recently, we discovered that G protein βγ subunits (Gβγ) interact with DAT, and that in vitro activation of Gβγ promotes DAT-mediated efflux. Here, we investigated the role of Gβγ in the actions of amphetamine in DA neurons in culture, ex vivo nucleus accumbens (NAc), and freely moving rats. Activation of Gβγ with the peptide myr-Ser-Ile-Arg-Lys-Ala-Leu-Asn-Ile-Leu-Gly-Tyr-Pro-Asp-Tyr-Asp (mSIRK) in the NAc potentiated amphetamine-induced hyperlocomotion, but not cocaine-induced hyperlocomotion, and systemic or intra-accumbal administration of the Gβγ inhibitor gallein attenuated amphetamine-induced, but not cocaine-induced hyperlocomotion. Infusion into the NAc of a TAT-fused peptide that targets the Gβγ-binding site on DAT (TAT-DATct1) also attenuated amphetamine-induced but not cocaine-induced hyperlocomotion. In DA neurons in culture, inhibition of Gβγ with gallein or blockade of the Gβγ–DAT interaction with the TAT-DATct1 peptide decreased amphetamine-induced DA efflux. Furthermore, activation of Gβγ with mSIRK potentiated and inhibition of Gβγ with gallein reduced amphetamine-induced increases of extracellular DA in the NAc in vitro and in freely moving rats. Finally, systemic or intra-accumbal inhibition of Gβγ with gallein blocked the development of amphetamine-induced, but not cocaine-induced place preference. Collectively, these results suggest that interaction between Gβγ and DAT plays a critical role in the actions of amphetamine and presents a novel target for modulating the actions of amphetamine in vivo.

## Introduction

Dopamine (DA) is a neuromodulator involved in motor, cognitive, motivational, and several other brain functions^1–3^. Abnormal dopaminergic signaling has been linked to several neurological disorders, including Parkinson’s disease, attention deficit/hyperactivity disorder (ADHD), depression, obsessive–compulsive disorder (OCD), and drug addiction^3–5^. Amphetamines are used in the treatment of many of these disorders but also are abused because of their reinforcing properties^6–8^. The ability of amphetamines to increase extracellular DA levels in the mesolimbic dopaminergic system, particularly in the nucleus accumbens (NAc), plays an important role in their addictive properties^6–8^.

Cocaine, another psychostimulant, also increases brain extracellular DA, but the mechanisms of action of cocaine and amphetamines differ^9–11^. Cocaine increases extracellular DA levels by blocking DA reuptake via the DA transporter (DAT)^12–14^. In contrast, amphetamines are competitive substrates of DAT that induce DA efflux through DAT^15,16^. The molecular mechanisms responsible for DA efflux by amphetamines have been the subject of intense investigation but still are not fully understood. Several proteins interact with and regulate DA efflux through DAT. Among these are protein kinase C (PKC), Ca^2+^/calmodulin-dependent protein kinase II (CAMKII), and syntaxin 1A^17–22^. Using a proteomic approach, we recently identified the G protein βγ subunit (Gβγ) as a DAT interacting protein^23^. Specifically, using cultured cells we discovered a direct interaction between Gβγ and the carboxy terminus of DAT^23,24^. We showed in both heterologous cells and striatal tissue that activation of Gβγ with the peptide mSIRK (myristoylated Gβγ-binding peptide) reduces DAT-mediated DA uptake, and that this reduction does not result from alterations in cell surface expression of DAT^23^. More importantly, we demonstrated that Gβγ activation leads to DAT-mediated DA efflux in cells in culture in a fashion similar to amphetamine-induced DA efflux^24^. Taken together, these findings suggest that Gβγ activation and interaction with DAT is an endogenous signaling mechanism that mimics the actions of amphetamine. Therefore, the goal of the present study was to determine whether Gβγ interaction with DAT plays a role in the neurochemical and behavioral effects of amphetamine.

Along with elevating extracellular DA levels in the NAc and the dorsal striatum, administration of amphetamine was shown to increase locomotor activity^25–29^ as well as induce conditioned place preference^30^. Here, using pharmacological agents targeting Gβγ activation or the interaction between Gβγ and DAT, we found that Gβγ activation in the NAc potentiated the locomotor effect of amphetamine, and inhibition of Gβγ or its interaction with DAT in the NAc attenuated amphetamine-induced hyperlocomotion. In parallel, Gβγ activation in the NAc potentiated amphetamine-induced increases in extracellular DA, and Gβγ inhibition decreased the effect of amphetamine on extracellular DA in dopaminergic cultures, the NAc, and freely moving rats. Furthermore, inhibition of Gβγ in the NAc blocked the development of amphetamine-conditioned place preference. In contrast to the modulation of amphetamine-induced behavioral effects, manipulation of Gβγ activation or interaction with DAT did not alter cocaine-induced locomotor activity or place preference. Our findings suggest a critical role for Gβγ in amphetamine-induced DA efflux via DAT and its behavioral consequences, and have implications for the development of new targets in the treatment of DA-related disorders.

## Materials and methods

All experiments were carried out in accordance with the University of Pittsburgh Institutional Animal Care and Use Committee (IACUC), the University of Florida IACUC, and the National Institutes of Health Guides for the Care and Use of Animals and most experiments have been described previously^23,24,31,32^. Detailed methods are provided in Supplementary Information.

## Results

### Activation of Gβγ subunits in the NAc potentiates and inhibition of Gβγ subunits in the NAc or systemically blunts amphetamine-induced hyperlocomotion

**Fig. 1:**
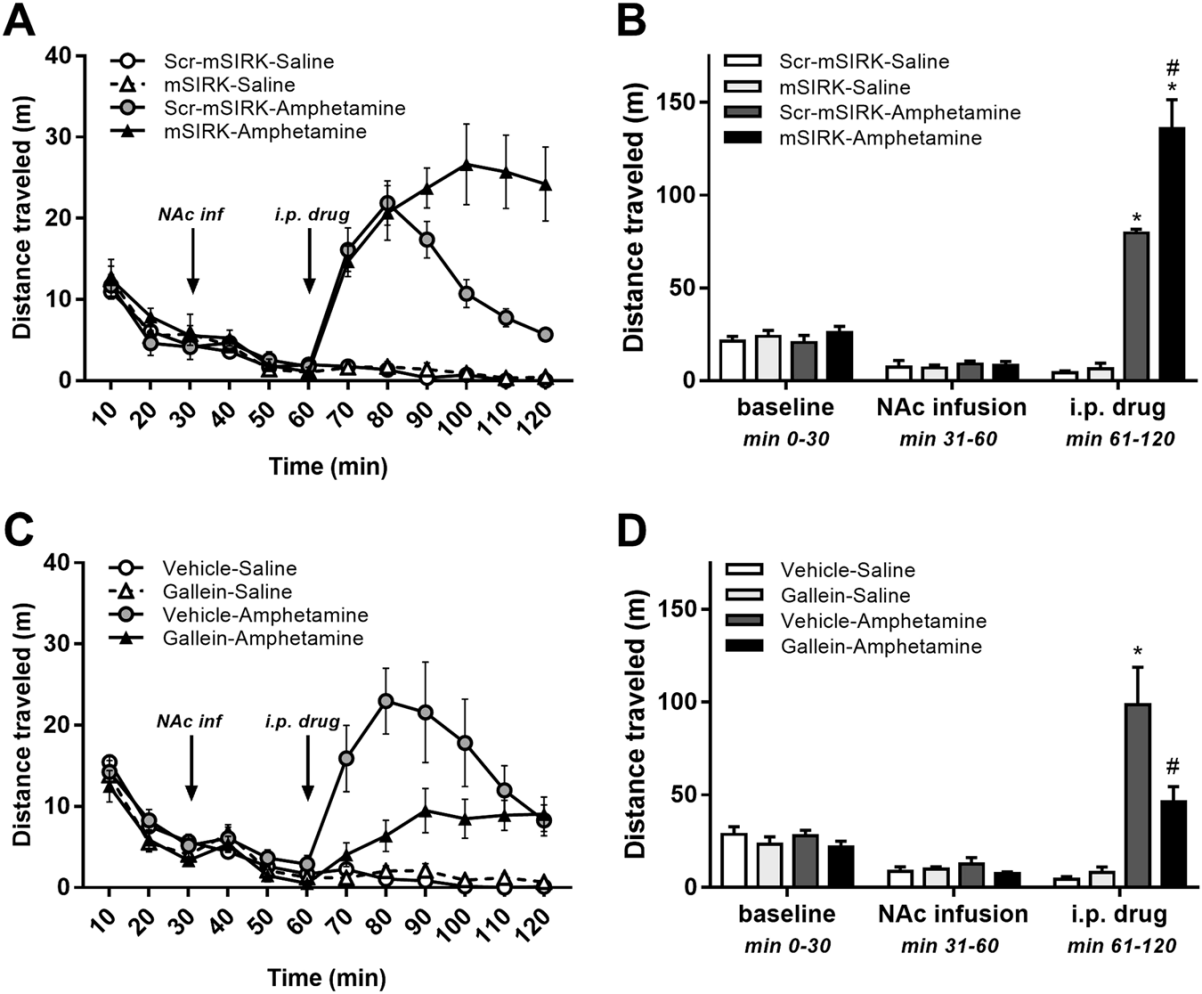
Activation of Gβγ subunits in the nucleus accumbens prolongs and inhibition of Gβγ subunits in the nucleus accumbens blunts amphetamine-induced hyperlocomotion. **a** Means ± s.e.m. of distance traveled across the 2 h testing period, depicted per 10 min block, for the scr-mSIRK-saline (*n* = 6), mSIRK-saline (*n* = 6), scr-mSIRK-amphetamine (*n* = 6) and mSIRK-amphetamine (*n* = 6) groups. Scr-mSIRK or mSIRK was infused into the nucleus accumbens (NAc) 30 min before saline or amphetamine injection. **b** Means ± s.e.m. of distance traveled by experimental period for the same groups. NAc infusion is the period immediately after intra-NAc infusion of scr-mSIRK, and intraperitoneal (i.p.) drug is the period immediately after saline or amphetamine injection. Habituation was performed in drug-free conditions. **c** Means ± s.e.m. of distance traveled across the 2 h testing period, depicted per 10 min block, for the vehicle-saline (*n* = 6), gallein-saline (*n* = 6), vehicle-amphetamine (*n* = 6) and gallein-amphetamine (*n* = 6) groups. Vehicle or gallein was infused into the NAc 30 min before saline or amphetamine injection. **d** Means ± s.e.m. of distance traveled by experimental period for the same groups. NAc infusion is the period immediately after intra-NAc infusion of vehicle of gallein, and i.p. drug is the period immediately after saline or amphetamine injection. Habituation was performed in drug-free conditions. *P < 0.05 between saline-treated and amphetamine-treated groups; ^#^*p* < 0.05 between scr-mSIRK-amphetamine and mSIRK-amphetamine groups **b** or between vehicle-amphetamine and gallein-amphetamine groups **d**

First, we investigated the effect of mSIRK, a cell-permeable myristoylated peptide that specifically activates Gβγ subunits^24,33,34^, delivered bilaterally into the NAc on basal and psychostimulant-induced locomotor activity. A scrambled sequence of mSIRK, scr-mSIRK, was used as control. As depicted in Fig. 1a, b, locomotor activity did not differ significantly between groups after bilateral infusion of mSIRK (1 mM) or scr-mSIRK (1 mM) into the NAc. After intraperitoneal psychostimulant injection, distance traveled was significantly higher for both groups injected with amphetamine (3 mg/kg) compared to the two groups injected with saline (Fig. 1a, b; *p* < 0.0001). Importantly, the amphetamine-induced hyperlocomotion in rats infused with mSIRK was extended markedly beyond the transient hyperlocomotion in rats infused with scr-mSIRK (1 mM), resulting in a significant potentiation of the psychostimulant effect (Fig. 1a, b; *p* < 0.001).

Next, we determined whether intra-accumbal infusion of the Gβγ inhibitor gallein, a small molecule that binds to Gβγ and disrupts Gβγ signaling to effectors^34–36^, affects basal and psychostimulant-induced locomotor activity. Similar to our observations above, locomotor activity did not differ significantly between groups during baseline or after bilateral infusion of gallein (2 mM) or vehicle into the NAc (Fig. 1c, d). After drug injection, distance traveled once again was significantly higher in amphetamine-injected compared to saline-injected rats (*p* < 0.001), but the magnitude of the hyperlocomotion was blunted markedly for rats that had received infusion of gallein compared to rats infused with vehicle (*p* < 0.02; Fig. 1c, d).

To investigate the translational possibilities of targeting Gβγ to modulate the effect of amphetamine, we repeated the previous experiment, but administered gallein intraperitoneally (4 mg/kg). Similar to our findings when the administration of the Gβγ inhibitor was restricted to the NAc, systemic gallein treatment had no effect on basal locomotor activity, but it significantly attenuated the elevation in locomotor activity after amphetamine injection (*p* < 0.0001; Figure S1). Taken together, our findings show that manipulation of Gβγ activity in the NAc modulates amphetamine’s locomotor effects bi-directionally, and that systemic administration of the Gβγ inhibitor gallein is as effective in blunting amphetamine-induced hyperlocomotion as is intra-NAc administration.

### Cocaine-induced hyperlocomotion is not affected by activation or inhibition of Gβγ subunits in the NAc

**Fig. 2:**
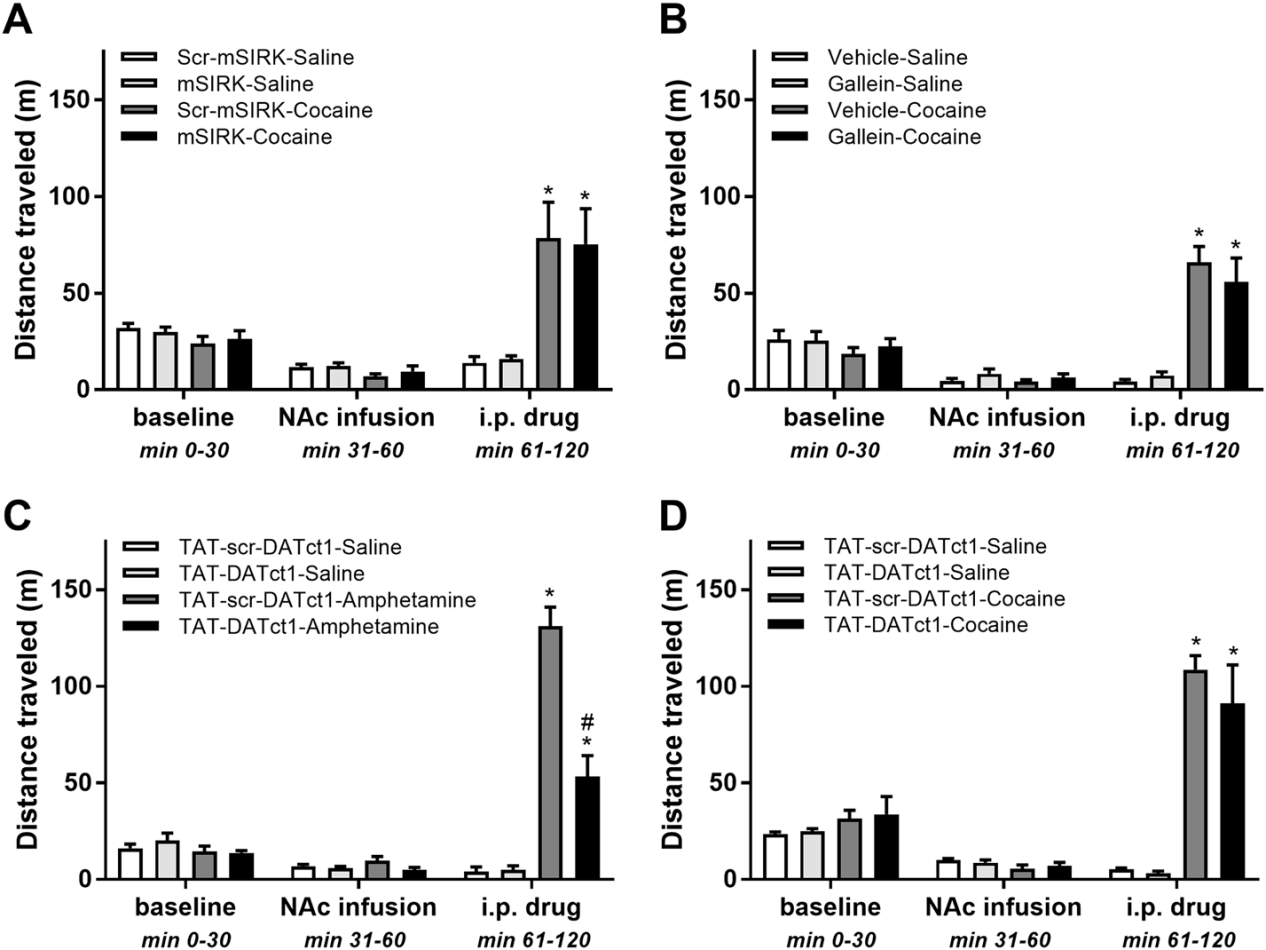
Activation or inhibition of Gβγ subunits in the nucleus accumbens has no effect on cocaine-induced hyperlocomotion, whereas interference of Gβγ subunit binding with dopamine transporter (DAT) in the nucleus accumbens attenuates amphetamine-induced locomotor activity. **a** Means ± s.e.m. of distance traveled by experimental period for the scr-mSIRK-saline (*n* = 6), mSIRK-saline (*n* = 6), scr-mSIRK-cocaine (*n* = 6), and mSIRK-cocaine (*n* = 6) groups. Nucleus accumbens (NAc) infusion is the period immediately after intra-NAc infusion of scr-mSIRK, and intraperitoneal (i.p.) drug is the period immediately after saline or amphetamine injection. Habituation was performed in drug-free conditions. **b** Means ± s.e.m. of distance traveled by experimental period for the vehicle-saline (*n* = 6), gallein-saline (*n* = 6), vehicle-cocaine (*n* = 6), and gallein-cocaine (*n* = 6) groups. NAc infusion is the period immediately after intra-NAc infusion of vehicle of gallein, and i.p. drug is the period immediately after saline or cocaine injection. Habituation was performed in drug-free conditions. **c** Means ± s.e.m. of distance traveled by experimental period for the TAT-scr-DATct1-saline (*n* = 5), TAT-DATct1-saline (*n* = 5), TAT-scr-DATct1-amphetamine (*n* = 6), and TAT-DATct1-amphetamine (*n* = 5) groups. NAc infusion is the period immediately after intra-NAc infusion of TAT-scr-DATct1 or TAT-DATct1, and i.p. drug is the period immediately after saline or amphetamine injection. Habituation was performed in drug-free conditions. **d** Means ± s.e.m. of distance traveled by experimental period for the TAT-scr-DATct1-saline (*n* = 5), TAT-DATct1-saline (*n* = 5), TAT-scr-DATct1-cocaine (*n* = 5), and TAT-DATct1-cocaine (*n* = 5) groups. NAc infusion is the period immediately after intra-NAc infusion of TAT-scr-DATct1 or TAT-DATct1, and i.p. drug is the period immediately after saline or cocaine injection. Habituation was performed in drug-free conditions. *P < 0.05 between saline-treated and cocaine- or amphetamine-treated groups; ^#^*p* < 0.05 between TAT-scr-DATct1-amphetamine and TAT-DATct1-amphetamine groups

As a first step toward deciphering the mechanism through which manipulation of Gβγ subunits in the NAc modulates amphetamine-induced hyperlocomotion, we determined whether cocaine-induced hyperlocomotion is also sensitive to pharmacological manipulation of intra-NAc Gβγ with mSIRK or gallein. If the effects of manipulating Gβγ on amphetamine-induced hyperlocomotion are related to amphetamine’s ability to induce DA efflux through DAT, then these same Gβγ manipulations should not affect cocaine-induced locomotor activity. Figure 2a depicts that, as observed previously, infusion of mSIRK (1 mM) or scr-mSIRK (1 mM) had no effect on locomotor activity before psychostimulant injection. After drug injection, locomotor activity was significantly higher in both groups that received cocaine (20 mg/kg, intraperitoneally (i.p.)) than in their saline-injected counterparts (*p* < 0.0001). However, unlike our observations with amphetamine, the magnitude as well as the time course of the psychostimulant-induced hyperlocomotion was unaffected by mSIRK (Fig. 2a). A similar experiment with intra-NAc infusion of gallein (2 mM) replicated the lack of an effect of the Gβγ inhibitor on basal activity, and, importantly, on cocaine-induced hyperlocomotion (Fig. 2b). The differential effect of Gβγ manipulation on hyperlocomotion induced by amphetamine vs. cocaine is consistent with the hypothesis that the underlying mechanism of the modulatory effect involves amphetamine’s ability to cause DA efflux via DAT.

### Blocking Gβγ interaction with DAT in the NAc blunts amphetamine-induced but not cocaine-induced hyperlocomotion

To elucidate further the mechanism through which Gβγ inhibition modulates amphetamine’s locomotor effects, we examined whether amphetamine-induced hyperlocomotion involves a direct interaction between Gβγ and the carboxy terminus of DAT. We previously showed that residues 582 to 596 in the carboxy terminus of DAT bind directly to Gβγ, and that a TAT-fused peptide containing these residues in DAT, TAT-DATct1, prevented Gβγ–DAT interaction^24^. Therefore, we examined the effect of bilateral intra-NAc infusion of TAT-DATct1 on amphetamine-induced hyperlocomotion. A scrambled sequence of DATct1 fused to TAT, TAT-scr-DATct1, served as control. Similar to the observations with gallein, intra-NAc infusion of TAT-DATct1 (1 mM) had no effect on basal locomotor activity but markedly attenuated the magnitude of the increase in locomotor activity induced by amphetamine (Fig. 2c). Although higher than in saline-injected rats, distance traveled was significantly lower for amphetamine-injected rats that had received intra-NAC infusion of TAT-DATct1 compared to rats that had received intra-NAc infusion of TAT-scr-DATct1 (Fig. 2c; *p* < 0.0001). In sharp contrast, intra-NAc infusion of TAT-DATct1 vs. TAT-scr-DATct1 did not result in differential effects on elevated locomotor activity induced by cocaine (Fig. 2d). Immunoprecipitation of DAT did not result in the co-precipitation of Gβγ in tissue from animals receiving TAT-DATCt1 compared to control conditions (data not shown). Taken together with our findings above, these results support a role for Gβγ interaction with DAT in amphetamine-induced hyperlocomotion.

### Inhibition of Gβγ activation or interaction with DAT decreases amphetamine-stimulated DA efflux in dopaminergic neurons in culture

**Fig. 3:**
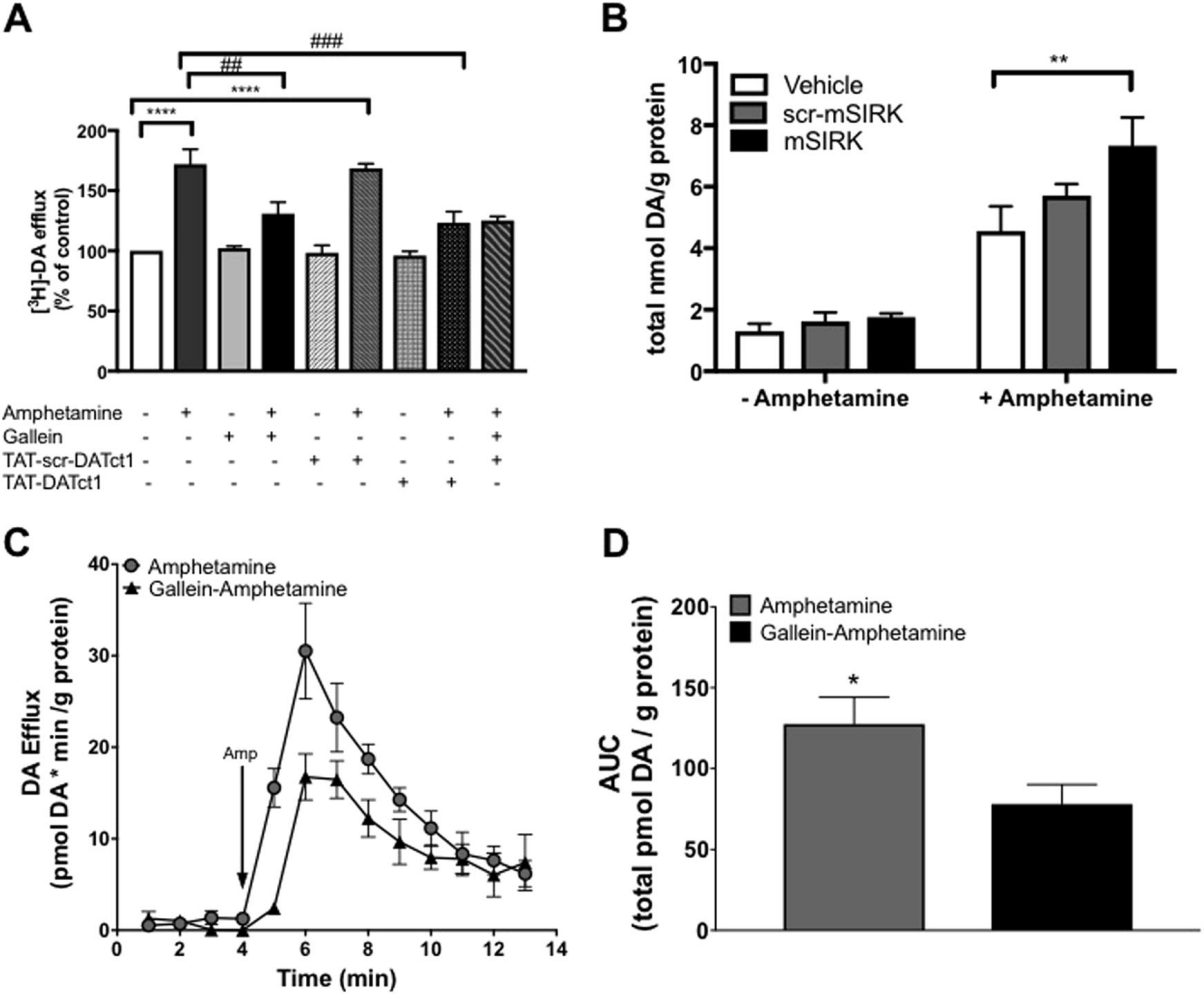
Activation or inhibition of Gβγ subunits or Gβγ–DAT interaction alters amphetamine-induced dopamine (DA) efflux in DA neurons in culture and nucleus accumbens tissue. **a** Inhibition of Gβγ subunits or Gβγ–DAT interaction blunts amphetamine-induced DA efflux in DA neurons preloaded with 20 nM^3^[H]-DA. Data represented as percent of control conditions for neurons treated with gallein (20 μM), TAT-scr-DATct1 (20 μM), TAT-DATct1 (20 μM), or TAT-DATct1 (20 μM) plus gallein (20 μM), ±amphetamine (10 μM) (*n* = 5/group). **b** Activation of Gβγ subunits increases amphetamine-induced DA efflux in nucleus accumbens (NAc) tissue. Mean ± s.e.m. of total DA efflux for tissue treated with vehicle (*n* = 8), scr-mSIRK (100 μM) (*n* = 10), or mSIRK (100 μM) (*n* = 10) before and after amphetamine (10 μM). **c** Inhibition of Gβγ subunits reduces amphetamine-induced DA efflux. Fractional release of the total DA of tissue perfused with amphetamine alone (10 μM) compared to gallein (20 μM)+amphetamine (10 μM) (*n* = 5/group). **d** Area under the curve (AUC) data for total DA efflux of tissue perfused with amphetamine alone compared to gallein+amphetamine.*P < 0.05 between amphetamine and gallein-amphetamine in NAc tissue. **p < 0.01 between mSIRK-amphetamine and vehicle-amphetamine in NAc tissue; ^##^*p* < 0.01 between amphetamine and gallein-amphetamine in DA neurons, ^###^*p* < 0.001 between amphetamine and TAT-DATct1-amphetamine in DA neurons, ****p < 0.0001 between control and amphetamine or scr-TAT-DATct1-amphetamine in DA neurons

The behavioral findings with the TAT-DATct1 peptide prompted us to investigate the role of Gβγ–DAT interaction in amphetamine-stimulated DA efflux in more depth. First, we examined the effects of the Gβγ inhibitor gallein and the TAT-DATct1 peptide on amphetamine-induced DA efflux in midbrain DA neurons in culture. Neurons were preloaded with [^3^H]-DA (20 nM) prior to treatment, and [^3^H]-DA release was assessed before and after amphetamine exposure. Amphetamine (10 μM) significantly increased DA efflux over control conditions (*p* < 0.0001; Fig. 3a). The effect of amphetamine on DA efflux was blunted in neurons pretreated with gallein (20 μM) (*p* < 0.01) or the TAT-DATct1 peptide (20 μM) (*p* < 0.001). The combined treatment of gallein and TAT-DATct1 peptide did not decrease further the amphetamine-induced efflux when compared to either gallein or TAT-DATct1 alone. Basal DA efflux was not affected by gallein, TAT-scr-DATct1, or TAT-DATct1 prior to amphetamine treatment, and the TAT-scr-DATct1 peptide had no effect on amphetamine-induced DA efflux (Fig. 3a). Thus, blocking Gβγ activation with gallein or blocking the Gβγ–DAT interaction with the TAT-DATct1 peptide reduces amphetamine-induced DA efflux.

### Activation of Gβγ with mSIRK potentiates and inhibition of Gβγ with gallein reduces amphetamine-induced DA efflux in dorsal striatum and NAc ex vivo

To assess further the role of Gβγ–DAT interaction in the actions of amphetamine, we tested the effect of Gβγ activation on amphetamine-induced DA efflux using an ex vivo DA efflux assay. DA efflux did not differ in the dorsal striatal punches treated with vehicle (1% dimethyl sulfoxide (DMSO)), scr-mSIRK (100 μM), or mSIRK (100 μM) during the 20-min pretreatment, prior to amphetamine treatment (10 μM) (Figure S2). However, amphetamine (10 μM) administration significantly increased DA efflux in samples pretreated with mSIRK compared to samples pretreated with vehicle or scr-mSIRK (*p* < 0.0001). As was observed with the dorsal striatum, there was no difference in NAc tissue treated with vehicle, scr-mSIRK, or mSIRK prior to amphetamine treatment (Fig. 3b). After amphetamine administration, there was a significant increase in DA efflux in mSIRK-treated tissue compared to vehicle-treated tissue (*p* < 0.001). In summary, activation of Gβγ with mSIRK increased DA efflux after amphetamine exposure in the dorsal striatum and the NAc ex vivo.

The effect of Gβγ inhibition with gallein on amphetamine-induced DA efflux was also examined in dorsal striatal and NAc tissue (Fig. S2 and 3c, d). Amphetamine (10 μM) alone caused a significant increase in DA efflux in dorsal striatal tissue. However, there was a significant reduction in DA efflux in tissue pretreated with gallein (20 μM) compared to tissue treated with amphetamine only (*p* < 0.05) (Figure S2). Similarly, in the NAc, there was a significant reduction in amphetamine-induced DA efflux with tissue treated with gallein prior to amphetamine compared to tissue treated with amphetamine alone (*p* < 0.05) (Fig. 3c, d). These data show that Gβγ inhibition reduces amphetamine-induced DA efflux in ex vivo dorsal striatum and NAc tissue.

### Activation of Gβγ with mSIRK potentiates and inhibition of Gβγ with gallein reduces amphetamine-induced increases in extracellular DA in the NAc of freely moving rats

**Fig. 4:**
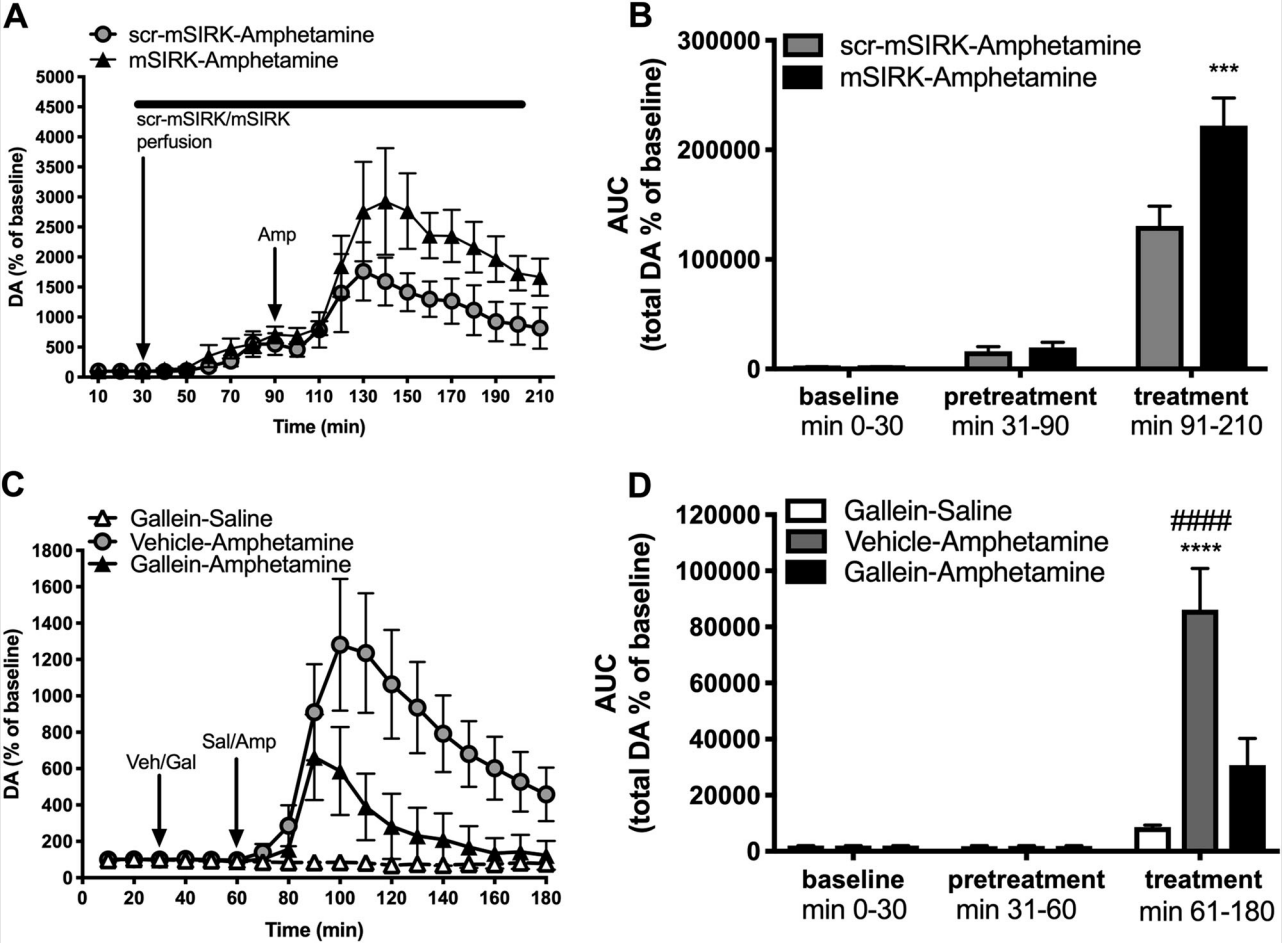
Activation or inhibition of Gβγ subunits alters amphetamine-induced extracellular dopamine (DA) levels in the nucleus accumbens of freely moving rats. **a** Activation of Gβγ subunits increases amphetamine-induced extracellular DA levels in the nucleus accumbens (NAc). Data represented as percent increase of baseline extracellular DA levels per 10 min for rats treated with scr-mSIRK (1 mM)–amphetamine (3 mg/kg) (*n* = 4) or mSIRK (1 mM)–amphetamine (3 mg/kg) (*n* = 5) over 210 min of testing. scr-mSIRK or mSIRK was perfused through the microdialysis probe by reverse dialysis for 1 h (pretreatment period) before an intraperitoneal (i.p.) injection of amphetamine (3 mg/kg) (treatment period) and continually perfused through the 2 h treatment period. **b** Area under the curve (AUC) of total extracellular DA levels during the baseline, pretreatment, and treatment periods. **c** Inhibition of Gβγ subunits reduces amphetamine-induced extracellular dopamine levels in the NAc of freely moving rats. Data represented as percent increase of baseline extracellular DA levels per 10 min for rats treated with gallein (4 mg/kg)–saline (1 ml/kg) (*n* = 4), vehicle (25% dimethyl sulfoxide (DMSO) in saline)–amphetamine (3 mg/kg) (*n* = 7) or gallein–amphetamine (*n* = 7) over the 180 min testing period. The i.p. injections of vehicle or gallein occurred during the pretreatment period followed by an i.p. injection of amphetamine or saline during the 2 h treatment period. **d** AUC analysis of total extracellular DA levels during the baseline, pretreatment, and treatment periods. ***P < 0.001 denotes AUC between scr-mSIRK-amphetamine and mSIRK-amphetamine during the treatment period, ****p < 0.0001 denotes AUC between gallein-saline and vehicle-amphetamine during the treatment period, ^####^*p* < 0.0001 denotes AUC between vehicle-amphetamine and gallein-amphetamine during the treatment period

Next, we determined the effects of Gβγ manipulation on amphetamine-induced increases in extracellular DA in the NAc of awake, freely moving rats using in vivo microdialysis. First, we assessed the effects of Gβγ activation with mSIRK on amphetamine-stimulated increase in extracellular DA. There was no difference in extracellular DA levels between groups during either the baseline period or the scr-mSIRK vs. mSIRK (1 mM) pretreatment period, i.e., prior to amphetamine (3 mg/kg) administration (Fig. 4a, b). However, amphetamine induced a significant increase in DA overflow in rats pretreated with mSIRK compared to rats pretreated with the control peptide scr-mSIRK (*p* < 0.001) (Fig. 4a, b). The present results show that Gβγ activation potentiates amphetamine’ s effects on DA overflow in the NAc of awake rats.

We also tested the effect of Gβγ inhibition on amphetamine-stimulated increases in extracellular DA. Again, there was no difference in extracellular DA levels during the baseline period, or the gallein (4 mg/kg) vs. vehicle pretreatment period (Fig. 4c, d). During the drug treatment period, amphetamine (3 mg/kg) significantly increased extracellular DA levels in rats that had received vehicle pretreatment, and the amphetamine-induced increase in extracellular DA was blunted in rats pretreated with gallein (*p* < 0.0001) (Fig. 4c, d). Thus, inhibition of Gβγ activation with gallein significantly reduced amphetamine-stimulated extracellular DA in awake rats. This outcome is consistent with the effect of gallein on DA efflux in ex vivo tissue samples and on amphetamine-induced locomotor activity.

### Inhibition of Gβγ subunits in the NAc blunts amphetamine-conditioned place preference but has no effect on cocaine-conditioned place preference

**Fig. 5:**
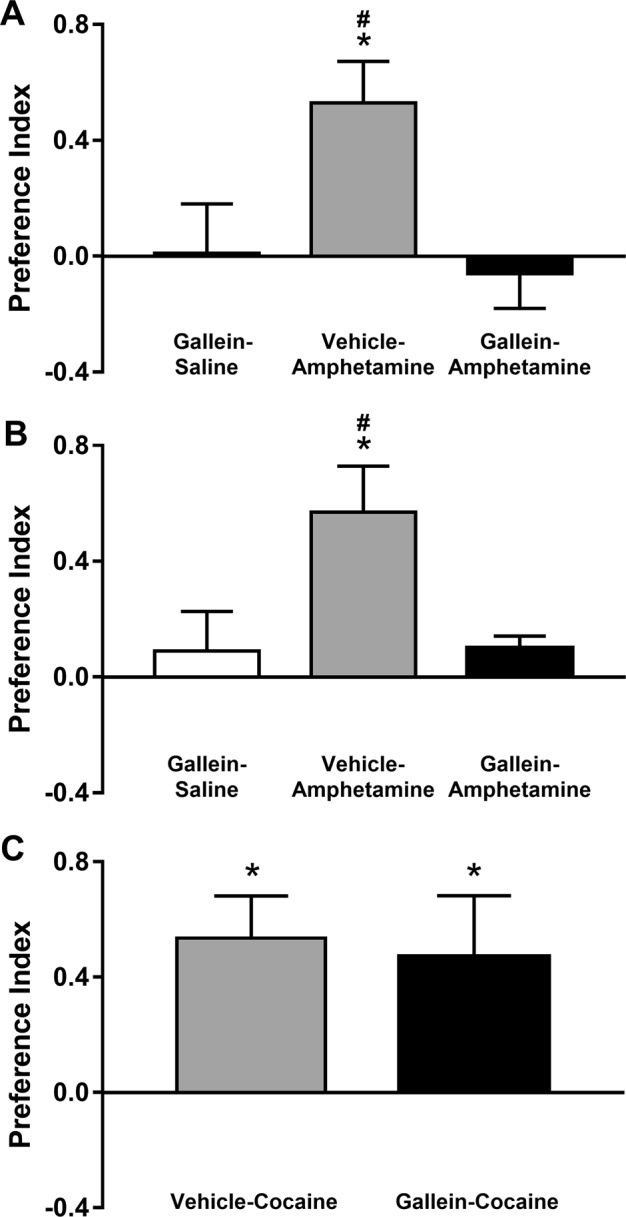
Inhibition of Gβγ subunits in the nucleus accumbens blunts amphetamine-induced place preference but has no effect on cocaine-induced place preference. **a** Means ± s.e.m. of conditioned place preference index for the gallein-saline (*n* = 9), vehicle-amphetamine (*n* = 10), and gallein-amphetamine (*n* = 10) groups. Vehicle or gallein was infused directly into the nucleus accumbens (NAc) 30 min before saline or amphetamine injection (intraperitoneally (i.p.)) on days 1, 3, and 5. **b** Similar data as shown in **a**, except that vehicle or gallein was administered i.p. 30 min before saline or amphetamine injection. The group sizes were identical to those in **a**. Animals that received gallein, whether by intra-NAc infusion (**a**) or i.p. injection (**b**), displayed neither a place preference nor an aversion, regardless of whether they underwent amphetamine conditioning or served as gallein-only controls. **c** Means ± s.e.m. of the conditioned preference index for cocaine-treated animals (*N* = 7 for both groups). Vehicle or gallein was administered i.p. 30 min before cocaine injection (i.p.) on days 1, 3, and 5. Animals displayed a place preference for the cocaine-paired compartment, regardless of whether they were pretreated with gallein or vehicle. *P < 0.05 between group average and 0.0 (no conditioning-induced shift in preference); ^#^*p* < 0.05 between the vehicle-amphetamine-treated group and either of the two gallein-pretreated groups

To determine whether the modulation of amphetamine’s neurochemical and locomotor effects by manipulation of Gβγ activity or its interaction with DAT in the NAc extends to the rewarding effects of amphetamine, we investigated the effect of bilateral intra-NAc infusion of gallein on amphetamine-mediated conditioned place preference. As shown in Fig. 5a, rats that received intra-NAc infusions of vehicle solution before amphetamine (1.5 mg/kg, i.p.) conditioning trials exhibited a significant, robust preference above pre-conditioning level for the amphetamine-paired compartment (*p* < 0.005). In contrast, rats that received intra-accumbal infusion of gallein (2 mM) before amphetamine conditioning trials displayed neither a preference for nor an aversion of the amphetamine-paired compartment. The lack of the amphetamine-conditioned place preference in the latter group did not appear to stem from aversive effects of gallein, because gallein alone had no effect on place conditioning, as indicated by the absence of conditioned place preference or aversion by rats that received intra-accumbal infusion of gallein in the absence of amphetamine conditioning trials (saline conditioning trials only, Fig. 5a).

To determine whether these observations extend to conditions with relatively greater translational relevance, we repeated the same experiment described above, but administered gallein systemically before drug conditioning trials. Once again, amphetamine-conditioned rats pretreated with vehicle solution developed a significantly positive preference index (Fig. 5b; *p* < 0.01), and, similar to our observations with gallein administration restricted to the NAc, rats that received systemic administration of gallein (4 mg/kg, i.p.) before amphetamine conditioning trials failed to develop an increase in preference for the amphetamine-paired compartment (Fig. 5b). Repeated exposure to systemic gallein alone did not produce an aversion, as indicated by a preference index near 0.0 by the gallein-treated group that received saline trials only.

We reported above that gallein or the TAT-DATct1 peptide had no effect on cocaine-induced hyperlocomotion, an outcome that suggested that Gβγ modulates amphetamine’s actions on DA efflux via DAT. To test whether the differential effect of Gβγ inhibition on amphetamine- vs. cocaine-induced locomotor behavior also extends to psychostimulant-induced reward seeking, we exposed rats to cocaine place preference conditioning using essentially the same conditioning paradigm, except that the experiment entailed only two groups, cocaine-conditioned rats pretreated with vehicle solution and cocaine-conditioned rats pretreated with gallein on psychostimulant conditioning days. Figure 5c shows that both vehicle- and gallein-pretreated rats developed a robust preference for the cocaine-paired compartment (both *p* < 0.05), and that the level of place preference did not differ between the two groups. Taken together, these results demonstrate that Gβγ inhibition, with gallein within the NAc or systemically, specifically modulates not only amphetamine-induced locomotor activity, but also amphetamine-conditioned place preference, an indicator of reward seeking.

## Discussion

Previously, we demonstrated that Gβγ subunits regulate DAT activity in heterologous cell lines and DA neurons in culture^23,24^, and established that Gβγ activation promotes DAT-mediated DA efflux in a manner that resembles amphetamine actions at DAT^24^. Here, we investigated whether Gβγ subunits are involved in the actions of amphetamine. Specifically, we examined the effect of Gβγ manipulation on amphetamine-induced behaviors, as well as DA overflow in neurons, striatal, and NAc tissue, and in the NAc of awake rats. Activation of Gβγ subunits in the NAc potentiated the effects of amphetamine on locomotor activity, and intra-NAc inhibition of Gβγ subunits reduced amphetamine-induced locomotor activity. Blocking Gβγ interaction with DAT with the TAT-DATct1 peptide similarly reduced amphetamine-induced locomotor activity, and intra-NAc or systemic inhibition of Gβγ subunits prevented the development of amphetamine-conditioned place preference. In addition, inhibition of Gβγ subunits decreased amphetamine-induced DA efflux in neurons in culture as well as in ex vivo striatal and NAc tissue, and it blunted amphetamine-induced increases in extracellular DA levels in the NAc of awake rats. Taken together, our data suggest that Gβγ subunits play an important role in the mechanism by which amphetamine increases extracellular DA and locomotor activity, and supports conditioning of place preference.

Different from the effects of amphetamine, cocaine-induced locomotor activity and conditioned place preference was insensitive to manipulation of Gβγ activity or disruption of the DAT- Gβγ interaction. This marked difference renders it unlikely that Gβγ subunits act through mechanisms involving postsynaptic DA receptors, or through a presynaptic mechanism independent of DAT. Given the difference in mechanism by which amphetamine and cocaine cause elevation in extracellular DA and associated behavioral changes^14–16^, and our findings that blockade of direct interaction of Gβγ subunits with DAT recapitulates the effects of the Gβγ inhibitor gallein, the most parsimonious explanation of our findings is that manipulation of Gβγ modulated the effects of amphetamine by targeting DA efflux via DAT. Thus, we surmise that Gβγ subunits are involved in the regulation of DA efflux and play a pivotal role in the behavioral and neurochemical effects of amphetamine through their interaction with DAT.

We previously observed an increase in DA efflux with Gβγ activation in cultured cells expressing DAT and preloaded with DA, an effect that was comparable to that of amphetamine^24^. Here, we found no effect of intra-NAc activation of Gβγ subunits with mSIRK on basal but only on amphetamine-induced elevated locomotor activity. Consistent with these behavioral results, we found that the main effect of mSIRK in brain tissue in vitro or in vivo was to potentiate amphetamine-stimulated DA efflux or overflow. We interpret the discrepancy between efflux of DA from cells in culture preloaded with DA and the lack of DA efflux after mSIRK in untreated native tissue to suggest that the action of Gβγ is at the level of DAT, promoting an efflux-like conformation. The measureable manifestation of this effect, however, requires not only a change in the conformation of DAT but also a source of DA to be released. In cells in culture preloaded with DA, mSIRK can readily release DA through DAT. In contrast, in native tissue in the absence of available DA, the effect of mSIRK would be minimal. Amphetamine induces vesicular release of DA^15^, thereby creating a condition of free DA akin to that of DA loaded into cells in culture. Thus, our results suggest that Gβγ effect on DAT function occurs downstream of vesicular release of DA by amphetamine, consistent with a direct effect on the transporter^24^. Together with our conclusions above, a model emerges in which Gβγ subunits acting downstream of DA release from vesicles in the presence of amphetamine modulate DA efflux via DAT and thereby play a pivotal role in the behavioral effects of amphetamine.

The molecular mechanisms involved in amphetamine-mediated DA efflux are not fully understood. Several proteins, including PKC, CaMKII, syntaxin, and phosphoinositide 3-kinase (PI3K), were reported to play a role in amphetamine-induced DA efflux^17–22,37^. For example, PKC was found to phosphorylate serine residues located in the amino terminal of DAT that are important for amphetamine-induced DA efflux^38^. CaMKIIα was shown to bind to the carboxy terminal region of DAT and phosphorylate its amino terminus region, and this effect was found to play a role in amphetamine-stimulated locomotor activation and DA efflux^18,39^. Phosphatidylinositol 4,5-biphosphate (PIP2) is another molecule found to interact with DAT and modulate amphetamine-induced DA efflux^40^. PIP2 directly binds to the amino terminus of DAT and facilitates amphetamine-mediated DA efflux and amphetamine-induced locomotion in drosophila. The serotonin transporter also binds PIP2, and approaches that deplete PIP2 levels from the cell membrane result in reduced amphetamine-induced serotonin efflux^41^. The observations with PIP2 suggest a common mechanism for monoamine transporters. Interestingly, the activity of several ion channels was also found to be regulated by direct interactions with PIP2^42–49^. Modulation of channel activity by Gβγ subunits was shown to depend on the presence of PIP2 as demonstrated for the atrial K_Ach_^50^, voltage-gated calcium Ca_v_2.1^51^, and voltage-gated potassium Kv7.4 channel^52^. These studies suggest that Gβγ and PIP2 work in concert to modulate the activity of not only ion channels but also monoamine transporters. Our results show that, in addition to molecules previously identified to be important for amphetamine-induced DA efflux, the interaction of Gβγ subunits with DAT is also involved in amphetamine’s mechanism of action. We recently found that inhibition of mitogen-activated protein kinase (MAPK), PI3K, or PKC signaling pathways did not alter Gβγ-induced DA efflux in cells in culture^24^, which suggests that these protein kinases are not involved in the Gβγ–DAT interaction. However, it is possible that the Gβγ–DAT interaction is a part of a larger complex involving other proteins known to regulate amphetamine-induced DA efflux in vivo, especially considering that Gβγ inhibition with gallein resulted in a partial, but not complete, blockade of amphetamine-induced hyperlocomotion and DA efflux. Future studies examining the role of other protein–protein interactions or additional molecules, such as PIP2, that may play a role in the contribution of the Gβγ–DAT interaction to amphetamine’s effects are needed. Given that Gβγ subunits are associated with G protein-coupled receptors (GPCRs), additional studies investigating potential GPCRs involved in the contribution of Gβγ–DAT interaction to DA efflux caused by amphetamine are warranted as well.

The behavioral manifestations associated with amphetamine’s actions are the result of complex and not completely understood mechanisms, including inhibition of DAT-mediated uptake, stimulation of DAT-mediated efflux, as well as internalization of DAT from the cell surface. In this context, the effect of amphetamine on DAT is analogous to the ligand-induced activation and subsequent internalization of some GPCRs. Several studies have provided compelling evidence for amphetamine-induced DAT internalization^53–55^, and the trafficking mechanisms involved in the internalization process are subject of intense investigation^56–58^. A clear picture is emerging now in which the initial interaction of amphetamine with DAT changes the activity of the transporter to promote DA efflux, and subsequent amphetamine exposure induces internalization of DAT from the plasma membrane. Although our results are consistent with an important role for Gβγ in DAT-mediated efflux, they do not rule out a potential contribution to trafficking mechanisms as well.

Identifying the molecular mechanism of action of amphetamines is important in the development of pharmacological agents to curtail excessive amphetamine use, as well as in the refinement of treatment approaches to OCD and ADHD, for which amphetamines are used therapeutically^59,60^. Our findings that intra-NAc and systemic administration of gallein did not induce place aversion but blocked or markedly curbed the development of amphetamine-conditioned place preference point to Gβγ subunits and their regulation of DAT function as a potential target. Additional studies, including examination of the role of Gβγ subunits and their interaction with DAT in amphetamine self-administration, are needed to determine whether Gβγ subunits are involved in the reinforcing aspects of the drug. Nevertheless, the current findings suggest that inhibition of Gβγ–DAT interaction may aid in the discovery and refinement of therapeutic treatments for conditions that involve therapeutic amphetamine use or amphetamine abuse and addiction.

## Supplementary information

Methods

Supplemental Figures S1 and S2

Supplemental Tables S1 - S8
