# Supplementary material for: G protein βγ subunits play a critical role in the actions of amphetamine
Source: Transl Psychiatry. 2019 Feb 11;9:81. doi: 10.1038/s41398-019-0387-8 (PMC6370791; doi:10.1038/s41398-019-0387-8)
Supplement: Supplementary file 1 — Methods [file 41398_2019_387_MOESM1_ESM.docx]

**MATERIALS AND METHODS**

**Animals**
All behavioral experiments used male Sprague-Dawley rats (Hilltop, PA, and Harlan, MD) and carried out in accordance with the University of Pittsburgh Institutional Animal Care and Use Committee (IACUC), the University of Florida IACUC, and the National Institutes of Health Guides for the Care and Use of Animals.

**Surgical Procedures**

*Intracranial cannulation and infusion*. Implantation of cannulae for behavioral experiments was conducted as described previously (Shiflett et al., 2008; Kirschmann et al., 2014). Briefly, rats were fitted bilaterally with stainless steel guide cannula aimed at a region dorsal of the NAc. For intra-NaC administration of reagents, two injection cannulae were lowered into the NAc beyond the tip of the guide cannulae and aimed at the border between the core and the shell of the NAc,. A 2-cm incision along the midline was made, and two holes were drilled into the skull according to the following coordinates: 1.2 mm anterior to bregma and ±1.5 mm from the midline (Paxinos and Watson rat brain atlas). A 26-gauge stainless steel guide cannula (6 mm long; Plastics One, Roanoke, VA) was lowered into each hole 5.4 mm ventral relative to the dural surface of the brain and affixed to the surface of the skull with cyanoacrylate glue and dental cement, and the incision around the dental cement crown was sutured closed. Stylets of equal length as the guide cannulae were inserted into the cannulae to prevent blockage. Rats were monitored for signs of distress twice daily and given liquid acetaminophen (300 mg/day; orally) for 2 days after surgery, and were allowed to recover for one week before the start of testing. Stained sections were evaluated for accuracy of probe placement. Only data from placements within the brain region of interest were used for further analysis Volumes of 0.5 μl/side of reagent or vehicle was delivered over a 1-min period.

*Microdialysis cannulation*. For microdialysis experiments, rats were anesthetized using oxygen containing 5% isoflurane, maintained at 2.5% isoflurane, and placed in a stereotaxic frame equipped with blunt ear bars (Stoelting). An incision was made along the midline, and a unilateral guide cannula (CMA-11 guide cannula; CMA Microdialysis, Solna, Sweden) was implanted into the NAc using the same coordinates as described above. Anchor screws were also implanted into the skull, and dental acrylic was used to keep the cannula, anchor screws, and a slotted screw in place to provide an anchor for the head tether during microdialysis (Perm Reline, Henry Schein Animal Health). The animals were maintained on meloxicam (2 mg/kg) for 2 days after surgery and were allowed to recover for at least one week prior to testing. **Behavioral Procedures**

All behavioral testing was conducted in the Rodent Behavior Analysis Core of the University of Pittsburgh Schools of Public Health.

*Open Field Test*. Locomotor activity was assessed in a standard open-field arena for a total of 120 min. After the first 30 min, rats received a bilateral intra-accumbal infusion of either mSIRK (myristoylated-SIRKALNILGPDYD), scr-mSIRK (myristoylated-SLYRLISLAPRGDYD), gallein (Sigma-Aldrich), vehicle, TAT-DATct1 peptide (GYGRKKRRQRRRG-SLPGSFREKLAYAIA), or TAT-scr-DATct1 peptide (GYGRKKRRQRRRG-PIESRSLALAAGYKF) (Millipore), and after the second 30 min, rats received i.p. injection of either amphetamine (Sigma-Aldrich) , cocaine (Sigma-Aldrich), or saline (see Suppl. Table 1 for groups, conditions, and n/condition). In some experiments, rats received i.p. injection of either gallein or vehicle instead of intra-accumbal infusion.

*Conditioned Place Preference*. Pre-conditioning preference was assessed for a 20-min baseline period during which rats had free access to the center and both distinctly textured and black-white patterned conditioning compartments. On Days 1, 3, and 5 of conditioning, rats received bilateral intra-accumbal infusion of either gallein or vehicle 30 min before i.p. injection of either amphetamine or saline and were placed immediately thereafter into one of the conditioning compartments for a 40-min conditioning period. On Days 2 and 4, rats received i.p. injection of saline and were placed into the other compartment for a 40-min conditioning period. Post-conditioning preference was assessed using the identical protocol as described for pre-conditioning assessment. In some additional experiments, rats received i.p. injection of either gallein or vehicle 30 min before i.p. injection of either amphetamine, cocaine, or saline on conditioning Days 1, 3, and 5 (see Suppl. Table 1).

**Preparation of primary DA neurons in culture**

Primary dopaminergic cultures were generated from embryonic male rats (E15-E16) by taking coronal sections of the mesencephalon containing the substantia nigra and the ventral tegmental area. Tissue sections were dissociated in a solution of papain (15 units/mL) with 0.45 mg/mL L-cysteine in dissociation media (in mM: 90 Na_2_SO_4_, 30 K_2_SO_4_, 5.8 MgCl_2_, 0.25 CaCl_2_, 10 HEPES, and 20 glucose, 0.5% phenol red, pH 7.4) for 20 min at 35 C^o^. After two washes, cells were mechanically dissociated in trituration medium containing 0.1% trypsin inhibitor, 0.1% bovine serum albumin, and 10 mM HEPES in basal medium Eagle (BME), and isolated by centrifugation in BME containing 1% trypsin inhibitor and 1% bovine serum albumin for 4 min at 12.5 g. The cells were plated at a density of 2500 cells/well onto glass coverslips pre-coated with 20 ng/L poly D-lysine and 2 ng/L laminin in BME supplemented with 0.225% D-glucose, 0.67 mM glutamine, 67 units/ml penicillin, 6.7 mg streptomycin, 0.5% N2 supplement, 2% rat serum, and 10 mM HEPES. Cultures were maintained at 37°C in a 5% CO_2_ incubator for seven days, with medium changes every two days. Samples were stained with tyrosine hydroxylase (TH) (AB159, Millipore) to verify the presence of DA neurons in the dissected regions of midbrain.

**[^3^H]-DA efflux in dopamine neurons**

Neurons in culture were plated on 24-well plates and loaded with 0.02 μM [^3^H]-(3,4-[7-3H] dihydroxyphenylethylamine) (DA, 34.8 Ci/mmol; PerkinElmer) for 20 min at 30 °C in assay buffer (130 mM NaCl, 1.3 mM KCl, 1.2 mM KH_2_PO_4_, 10 mM HEPES and 2.2 mM CaCl_2_, pH 7.4) containing 10 mM glucose, 0.1 mM pargyline, 1 mM tropolone, and 0.1 mM ascorbic acid. After loading, cells were washed twice with assay buffer. Following washes, the neurons were incubated with either gallein (20 μM), TAT-scr-DATct1 (20 μM), or TAT-DATct1 (20 μM) for 10 min. Neuronal cultures then were incubated with amphetamine (10 μM) for 10 min to induce [^3^H]-DA efflux. The released [^3^H]-DA was measured using a LS-Counter (Beckman Coulter). Efflux was expressed as percentage (%) increase relative to the control group.

***Ex vivo* Efflux Assay**

Adult male rats were decapitated under isoflurane and their brains were removed quickly. 400 μm brain slices containing the dorsal striatum and the NAc were obtained using a vibratome. Two mm micropunches from dorsal striatum or NAc were taken and incubated in ice-cold oxygenated Krebs-Henseleit buffer (KH buffer: 116 mM NaCl, 3 mM KCl, 1 mM MgSO4, 1.2 mM KH2PO4, 11 mM D-glucose, 0.4 mM ascorbic acid, and 25 mM NaHCO3). Micropunches from striatum or NAc were loaded onto GF/B Whatman filters, and the filters were placed into a superfusion chamber (Brandel suprafusion 1000 system, Gaithersburg, MD) and perfused at 37°C for 20 min with oxygenated KH buffer containing 1.8 mM CaCl2 and 0.01 mM pargyline. After the incubation period, tissue punches were incubated with either gallein (20 μM) in oxygenated KH buffer or oxygenated KH buffer only for an additional 20 min. Superfusate samples containing extracellular DA were collected at a flow rate of 1 ml/min with four 1-min collections prior to amphetamine administration. Tissue punches then were incubated with amphetamine (10 μM) for a 2-min period, followed by 1-min collections of superfusate for another 20 min. To determine the effects of the Gβγ activator mSIRK on amphetamine-induced DA efflux, 2 mm micropunches from dorsal striatal or NAc were pre-incubated with KH buffer containing calcium and pargyline in a 48-well plate under oxygen for 30 min at 37°C. Tissue punches then were moved to wells containing oxygenated KH buffer with vehicle (1% DMSO in KH buffer), mSIRK, or scr-mSIRK (100 μM in vehicle) added for an additional 20 min at 37°C. After the 20-min incubation, an aliquot of the vehicle-, the scr-mSIRK-, or the mSIRK-treated KH buffer taken from the punches was used to determine basal DA levels prior to amphetamine administration. Subsequently, amphetamine (10 μM) was added to the wells containing the tissue punches to determine DA efflux. Later, tissue punches were recovered and homogenized in KH buffer with 1% Triton-X 100 to determine total protein concentration using the Dc Protein assay (Bio-Rad Laboratories, Hercules, CA). Perchloric acid (0.1 N ) was added to the samples to preserve the DA content, and samples were placed in centrifugal filter tubes and centrifuged at 500 g for 5 min. Filtered samples were recovered, and dopamine content was determined using HPLC with electrochemical detection.

***In vivo* microdialysis**

The microdialysis probe (CMA-11 probe with a 2-mm membrane length, cutoff 6000 Da; CMA Microdialysis) was connected to a microdialysis pump (CMA 402 syringe pump; CMA Microdialysis) with FEP tubing and a Instech liquid swivel (Plymouth Meeting, PA). Perfusion fluid (147 mM NaCl, 2.7 mM KCl, 1.2 mM CaCl2, 0.85 mM MgCl2; from CMA) was perfused through the probe at a flow rate of 0.1 μl/min. Rats were anesthetized briefly with 3% isoflurane, and the microdialysis probe was implanted into the guide cannula within the NAc. After probe implantation, rats were allowed to acclimate to the testing chamber for at least 16 h while the probe was continuously perfused at 0.1 μl/min. The next day, the flow rate was increased to 1.5 μl/min for at least 1 h before the first sample was collected. The baseline collection period consisted of three 10 min baseline samples collected prior to any drug administration. To examine the effects of the Gβγ inhibitor gallein on amphetamine-induced DA overflow, rats received an i.p. injection of gallein (4 mg/kg in vehicle) or vehicle (25% DMSO in sterile saline; 1ml/kg) and samples were collected for another 30 min. Then, rats received an i.p. injection of amphetamine (3 mg/kg in sterile saline) or saline (1ml/kg), and samples were collected for an additional 120 min. For experiments determining the effect of the Gβγ activator mSIRK on amphetamine-induced DA overflow, mSIRK or scr-mSIRK, (1 mM mSIRK or scr-mSIRK in perfusion fluid containing 10% DMSO) was perfused through the probe by reverse dialysis for 1 h after the baseline collection period. Then, rats received an i.p. injection of amphetamine (3 mg/kg in sterile saline), and mSIRK or scr-mSIRK was reverse-dialysed continually through the probe during amphetamine administration and throughout the remainder of the 2 h collection period. All microdialysis samples were collected in HPLC vials containing 10 mM acetic acid, and were analyzed using HLPC with electrochemical detection.

**HPLC analysis of DA**

DA was quantified from microdialysis and superfusion samples using HPLC with electrochemical detection. Samples were run on a Decade amperometric HPLC system (Antec LC-EC system, Antec, Leyden BV, the Netherlands) using a reverse phase column (Acquity UPLC BEH C-18 1.7 μm, 1 x 100 mm, Waters Co., Miliford, MA) with an electrochemical detector (2 mm glassy carbon electrode, SenCell, Antec) set at a potential of 0.8 V. The mobile phase consisting of 100 mM phosphoric acid, 100 mM citric acid, 0.1 mM EDTA-Na_2_, 600 mg/l octanesulfphonic acid (pH 3.0), and 8% acetonitrile was set at a flow rate of 0.05 ml/min. Clarity software (Antec) was used to acquire and analyze the chromatographic data.

**Statistical Analysis**

For the Open Field experiments, total distance traveled was determined for each testing period (baseline, post intra-accumbal infusion, and post i.p. injection), and the resultant data were analyzed for each testing period separately using two-way analyses of variance (ANOVAs), with infusion and injection as between-subject factors. Post-hoc pairwise comparisons with Bonferroni corrections were applied where interactions were found to be statistically significant. For the Conditioned Place Preference experiments, the total time spent in each of the two conditioning compartments (drug-paired, saline-paired) was determined for the pre-conditioning session and the post-conditioning session, and a Preference Index was calculated according to the following formula: Preference Index = (Time spent in drug-paired compartment during post-conditioning session - Time spent in drug-paired compartment during pre-conditioning session) / Time spent in drug-paired compartment during pre-conditioning session. Values near zero indicate no change in preference from pre-conditioning level. The resultant data were analyzed using one-sample Student’s *t*-tests and assuming a population mean of 0.0. Comparisons between groups were conducted using one-way analyses of variance (ANOVAs) and post-hoc pairwise comparisons with Bonferroni corrections (amphetamine experiments) or a Student’s *t*-test for independent groups (cocaine experiment). [3H]-DA efflux data are presented as a percent change from control conditions and were analyzed using one-way ANOVA followed by Bonferroni multiple comparisons test for post-hoc comparisons. *Ex vivo* efflux data were analyzed using area under the curve (AUC) data with a Student’s *t*-test for independent groups (gallein experiments), and two-way repeated-measures ANOVA with Bonferroni’s multiple comparison test for post-hoc pairwise comparisons (mSIRK experiments). Microdialysis data are presented as percent change from the mean value of the three baseline samples with the area under the curve (AUC) data used and evaluated with two-way ANOVA and Bonferroni multiple comparisons test for post-hoc comparisons for each testing period (baseline, pretreatment, and i.p. treatment). For all statistical analyses, the alpha level was set to ≤ 0.05. Data in figures are presented as means ± SEMs. Power analysis was used to determine sample size. Animals were allocated randomly to the different experimental groups and measurements were made blind to experimental group.

**REFERENCES**

23. Garcia-Olivares J, Torres-Salazar D, Owens WA, Baust T, Siderovski DP, Amara SG, Zhu J, Daws LC, Torres GE. Inhibition of dopamine transporter activity by G protein βγ subunits. *PLoS One* 2013; **8**:e59788.

24. Garcia-Olivares J, Baust T, Harris S, Hamilton P, Galli A, Amara SG, Torres GE. Gβγ subunit activation promotes dopamine efflux through the dopamine transporter. *Mol Psychiatry.* 2017; **22**:1673-1679.

30. Shiflett MW, Martini RP, Mauna JC, Foster RL, Peet E, Thiels E. Cue-elicited reward-seeking requires extracellular signal-regulated kinase activation in the nucleus accumbens. *J Neurosci*2008; **28**:1434-43.

31. Kirschmann EK, Mauna JC, Willis CM, Foster RL, Chipman AM, Thiels E. Appetitive cue-evoked ERK signaling in the nucleus accumbens requires NMDA and D1 dopamine receptor activation and regulates CREB phosphorylation. *Learn Mem*2014; **21**:606-15.

32. Paxinos G, Watson C. The rat brain in stereotaxic coordinates. 6th. Boston: Academic Press; 2007.
