# Supplementary material for: G protein βγ subunits play a critical role in the actions of amphetamine
Source: Transl Psychiatry. 2019 Feb 11;9:81. doi: 10.1038/s41398-019-0387-8 (PMC6370791; doi:10.1038/s41398-019-0387-8)
Supplement: Supplementary file 2 — Supplemental Figures S1 and S2 [file 41398_2019_387_MOESM2_ESM.docx]

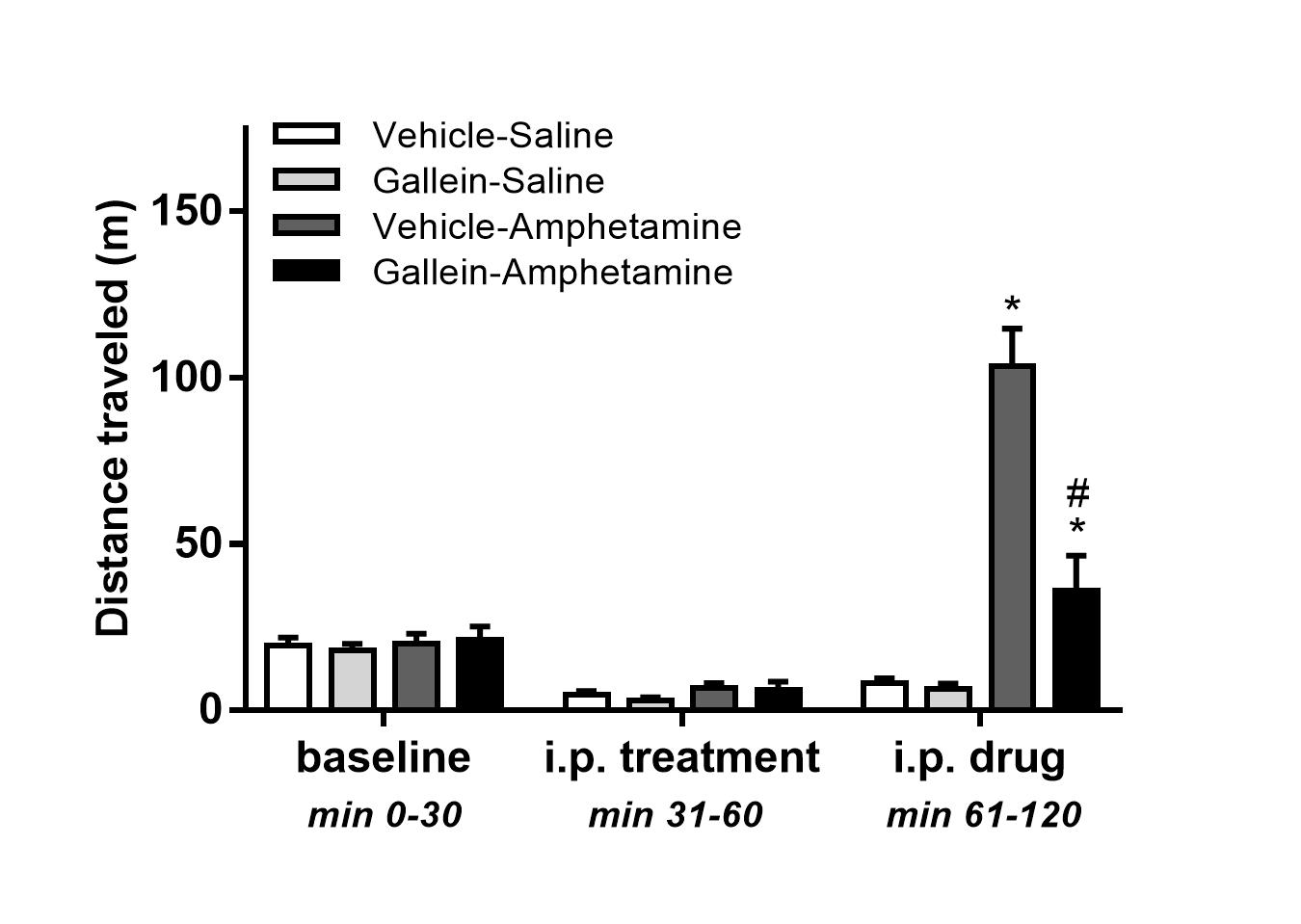


**Figure S1. Systemic inhibition of Gβγ subunits reduces amphetamine-induced locomotor activity.** Means ± s.e.m. of distance travelled by experimental period for the vehicle-saline (n=7), gallein-saline (n=7), vehicle-amphetamine (n=7) and gallein-amphetamine (n=7) groups. Vehicle or gallein was injected i.p. 30 min before saline or amphetamine injection. *i.p. treatment* is the period immediately after i.p. injection of vehicle or gallein, and *i.p. drug* is the period immediately after saline or amphetamine injection. Habituation was performed in drug-free conditions. * denotes p<0.05 between saline-treated and amphetamine-treated groups. # denotes p<0.05 between vehicle-amphetamine and gallein-amphetamine groups.

**Figure S2. Activation or inhibition of Gβγ subunits alters amphetamine-induced DA efflux in dorsal striatal tissue. A.** Activation of Gβγ subunits increases amphetamine-induced DA efflux in dorsal striatal tissue**.** Mean ± s.e.m of total extracellular DA for tissue treated with vehicle (n=10), scr-mSIRK (100 μM) (n=10) or mSIRK (100 μM) (n=11) before and after amphetamine treatment (10 μM). **B.** Inhibition of Gβγ subunits reduces amphetamine-induced DA efflux. Fractional release of extracellular DA of tissue perfused with amphetamine alone (10 μM) compared to gallein (20 μM) + amphetamine (10 μM) (n=5/group). **C.** AUC data for total DA efflux of tissue perfused with amphetamine alone compared to gallein + amphetamine. * denotes p<0.05 between amphetamine and gallein-amphetamine. **** denotes p<0.0001 between mSIRK-amphetamine compared to scr-mSIRK-amphetamine or vehicle-amphetamine.
