# Supplementary material for: G protein βγ subunits play a critical role in the actions of amphetamine
Source: Transl Psychiatry. 2019 Feb 11;9:81. doi: 10.1038/s41398-019-0387-8 (PMC6370791; doi:10.1038/s41398-019-0387-8)
Supplement: Supplementary file 3 — Supplemental Tables S1 - S8 [file 41398_2019_387_MOESM3_ESM.docx]

**Table S1.** Design of experiments examining the effect of Gβγ manipulation or Gβγ regulation of DAT (first column) on psychostimulant-induced locomotor activity, *ex vivo* and *in vivo* DA efflux, and conditioned place preference. Numbers in parentheses in bold indicate duration of the testing periods; numbers in parentheses after injected drug indicate sample size. See Methods for more details.

| Locomotor activity | | |
| --- | --- | --- |
| Intra-accumbal (30 min) | **Inject i.p.- Exptl (60 min)** | **Inject i.p.- Cntrl (60 min)** |
| mSIRK | amphetamine (6) | saline (6) |
| scr-mSIRK | amphetamine (6) | saline (6) |
| Gallein | amphetamine (6) | saline (6) |
| Vehicle | amphetamine (6) | saline (6) |
| mSIRK | cocaine (6) | saline (6) |
| scr-mSIRK | cocaine (6) | saline (6) |
| Gallein | cocaine (6) | saline (6) |
| Vehicle | cocaine (6) | saline (6) |
| TAT-DATct1 | amphetamine (5) | saline (5) |
| TAT-scr-DATct1 | amphetamine (6) | saline (5) |
| TAT-DATct1 | cocaine (5) | saline (5) |
| TAT-scr-DATct1 | cocaine (5) | saline (5) |
|  | | |
| Inject i.p. (30 min) | **Inject i.p.- Exptl (60 min)** | **Inject i.p.- Cntrl (60 min)** |
| Gallein | amphetamine (7) | saline (7) |
| Vehicle | amphetamine (7) | saline (7) |
|  | | |
| *Ex Vivo* DA efflux | | |
| Pretreatment (20 min) | **Exptl (5 min)** |  |
| mSIRK | amphetamine (11) |  |
| scr-mSIRK | amphetamine (10) |  |
|  | | |
| Pretreatment (20 min) | **Exptl (2 min)** |  |
| Gallein | amphetamine (5) |  |
| Vehicle | amphetamine (5) |  |
|  | | |
| *In vivo* Microdialysis | | |
| Pretreatment (60 min) | **Inject i.p.- Exptl (120 min)** |  |
| mSIRK | amphetamine (5) |  |
| scr-mSIRK | amphetamine (4) |  |
|  | | |
| Pretreatment (30 min) | **Inject i.p.- Exptl (120 min)** | **Inject i.p.- Cntrl (120 min)** |
| Gallein | amphetamine (7) | saline (4) |
| Vehicle | amphetamine (7) |  |
|  | | |
| Conditioned place preference | | |
| Intra-accumbal (30 min) | **Inject i.p.- Exptl (40 min)** | **Inject i.p.- Cntrl (40 min)** |
| Gallein | amphetamine (10) | saline (9) |
| Vehicle | amphetamine (10) |  |
| Gallein | cocaine (10) |  |
| Vehicle | cocaine (10) |  |
|  | | |
| Inject i.p. (30 min) | **Inject i.p.- Exptl (40 min)** | **Inject i.p.- Cntrl (40 min)** |
| Gallein | amphetamine (10) | saline (9) |
| Vehicle | amphetamine (10) |  |

**Table S2**. Statistical results for data depicted in Figure 1. * denotes statistically significant.

**Figure 1A (scr-mSIRK vs. mSIRK, saline vs. amphetamine)**

| baseline | infusion | | F1,20=1.478 | | p=0.238 |
| --- | --- | --- | --- | --- | --- |
|  | injection | | F1,20=0.038 | | p=0.845 |
|  | infusion x injection | | F1,20=0.215 | | p=0.647 |
| NAc infusion | infusion | | F1,20=0.067 | | p=0.798 |
|  | injection | | F1,20=0.443 | | p=0.513 |
|  | infusion x injection | | F1,20=0.002 | | p=0.960 |
| i.p. drug | infusion | | F1,20=13.12 | | p=0.0017* |
|  | injection | | F1,20=161.3 | | p=0.0001* |
|  | infusion x injection | | F1,20=11.22 | | p=0.0032* |
| **Figure 1B (vehicle vs gallein, saline vs. amphetamine, infusion)** | |  | |  |  |
| baseline | Infusion | | F1,20=2.426 | | p=0.1350 |
|  | Injection | | F1,20=0.1319 | | p=0.7203 |
|  | infusion x injection | | F1,20=0.0124 | | p=0.9125 |
| NAc infusion | Infusion | | F1,20=0.8096 | | p=0.3789 |
|  | Injection | | F1,20=0.0896 | | p=0.7678 |
|  | infusion x injection | | F1,20=2.130 | | p=0.1599 |
| i.p. drug | Infusion | | F1,20=4.883 | | p=0.039* |
|  | Injection | | F1,20=36.369 | | p<0.0001* |
|  | infusion x injection | | F1,20=6.500 | | p=0.0191* |

**Table S3.** Statistical results for data depicted in Figure S1. * denotes statistically significant.

**Figure S1 (vehicle vs. gallein i.p., saline vs. amphetamine)**

| baseline | Infusion | F1,24=0.0067 | p=0.9353 |
| --- | --- | --- | --- |
|  | Injection | F1,24=0.4939 | p=0.4889 |
|  | infusion x injection | F1,24=0.1849 | p=0.6711 |
| i.p. treatment | Infusion | F1,24=0.5827 | p=0.4527 |
|  | Injection | F1,24=2.936 | p=0.0995 |
|  | infusion x injection | F1,24=0.1565 | p=0.6959 |
| i.p. drug | Infusion | F1,24=23.18 | p<0.0001* |
|  | Injection | F1,24=75.75 | p<0.0001* |
|  | infusion x injection | F1,24=21.09 | p=0.0001* |

**Table S4.** Statistical results for data depicted in Figure 2. * denotes statistically significant.

**Figure 2A (scr-mSIRK vs. mSIRK, saline vs. cocaine)**

| baseline | Infusion | F1,20=0.0001 | p=0.9909 |
| --- | --- | --- | --- |
|  | Injection | F1,20=3.027 | p=0.0972 |
|  | infusion x injection | F1,20=0.4832 | p=0.4950 |
| NAc infusion | Infusion | F1,20=0.5417 | p=0.4703 |
|  | Injection | F1,20=3.141 | p=0.0916 |
|  | infusion x injection | F1,20=0.2154 | p=0.6476 |
| i.p. drug | Infusion | F1,20=0.0030 | p=0.9568 |
|  | Injection | F1,20=22.26 | p=0.0001* |
|  | infusion x injection | F1,20=0.03874 | p=0.8460 |

**Figure 2B (vehicle vs. gallein, saline vs. cocaine)**

| baseline | Infusion | F1,20=0.1466 | p=0.7058 |
| --- | --- | --- | --- |
|  | Injection | F1,20=1.518 | p=0.2322 |
|  | infusion x injection | F1,20=0.2932 | p=0.5942 |
| NAc infusion | Infusion | F1,20=2.519 | p=0.1282 |
|  | Injection | F1,20=0.4026 | p=0.5329 |
|  | infusion x injection | F1,20=0.1994 | p=0.6600 |
| i.p. drug | Infusion | F1,20=0.2079 | p=0.6533 |
|  | Injection | F1,20=52.8 | p<0.0001* |
|  | infusion x injection | F1,20=0.7685 | p=0.3911 |

**Figure 2C (scr-TAT-DATct1 vs. TAT-DATct1, saline vs. amphetamine)**

| baseline | Infusion | F1,17=0.3344 | p=0.5707 |
| --- | --- | --- | --- |
|  | Injection | F1,17=2.169 | p=0.1591 |
|  | infusion x injection | F1,17=0.7923 | p=0.3858 |
| NAc infusion | infusion | F1,17=2.68 | p=0.1200 |
|  | injection | F1,17=0.5259 | p=0.4782 |
|  | infusion x injection | F1,17=1.425 | p=0.2490 |
| i.p. drug | infusion | F1,17=21.99 | p=0.0002* |
|  | injection | F1,17=113.7 | p<0.0001* |
|  | infusion x injection | F1,17=22.94 | p=0.0002* |

**Figure 2D (scr-TAT-DATct1 vs. TAT-DATct1, saline vs. cocaine)**

| baseline | infusion | F1,16=0.1496 | p=0.7040 |
| --- | --- | --- | --- |
|  | injection | F1,16=2.696 | p=0.1201 |
|  | infusion x injection | F1,16=0. 0057 | p=0. 9406 |
| NAc infusion | infusion | F1,16=0.00003 | p=0.9957 |
|  | injection | F1,16=3.648 | p=0.0742 |
|  | infusion x injection | F1,16=0. 6435 | p=0. 4342 |
| i.p. drug | infusion | F1,16=0.8561 | p=0.3686 |
|  | injection | F1,16=81.22 | p<0.0001* |
|  | infusion x injection | F1,16=0.5262 | p=0.4787 |

**Table S5.** Statistical results for data depicted in Figure 3. * denotes statistically significant.

**Figure 3A (DA efflux in DA neurons, gallein, TAT-DATct1, amphetamine)**

| Treatment | F7,32=19.67 | P=0.0001* |
| --- | --- | --- |

**Figure 3B (DA efflux in Nac ex vivo, msirk)**

| Treatment | F2,25=4.413 | p=0.0288* |
| --- | --- | --- |
| Drug | F2,25=93.12 | p=0.0001* |
| Treatment x drug | F2,25=2.279 | p=0.1233 |

**Figure 3C (DA efflux in superfusion assay, NAc, gallein)**

| Veh-amp vs gal-amp | t(8)=2.41 | p=0.0425* |
| --- | --- | --- |

**Table S6.** Statistical results for data depicted in Figure S2. * denotes statistically significant.

**Figure S2A (DA efflux in DS ex vivo, msirk)**

| Treatment | F2,28=11.42 | p=0.0002* |
| --- | --- | --- |
| Drug | F2,28=131.2 | p=0.0001* |
| Treatment x drug | F2,28=5.989 | p=0.0068* |

**Figure S2B (DA efflux in superfusion assay, DS, gallein)**

| Veh-amp vs gal-amp | t(8)=2.455 | p=0.0396* |
| --- | --- | --- |

**Table S7.** Statistical results for data depicted in Figure 4. * denotes statistically significant.

**Figure 4A (microdialysis, mSirk)**

| period | F2,21=100 | p=0.0001* |
| --- | --- | --- |
| drug | F1,21=8.103 | p=0.0097* |
| period x drug | F2,21=7.197 | p=0.0042* |

**Figure 4B (microdialysis, gallein)**

| period | F2,45=31 | p=0.0001* |
| --- | --- | --- |
| drug | F2,45=10.38 | p=0.0002* |
| period x drug | F4,45=10.34 | p=0.0001* |

**Table S8.** Statistical results for data depicted in Figure 5. * denotes statistically significant.

**Fig 5A (vehicle vs. gallein intra-NAc, saline vs amphetamine)**

| gal-sal vs. 0 | t8=0.067 | p=0.9482 |
| --- | --- | --- |
| veh-amp vs. 0 | t9=3.7563 | p=0.0045* |
| gal-amp vs. 0 | t9=0.536 | p=0.6051 |
| infusion x injection | F1,35=6.722 | p=0.0138* |

**Fig 5B (vehicle vs. gallein i.p., saline vs amphetamine)**

| gal-sal vs. 0 | t8=0.961 | p=0.365 |
| --- | --- | --- |
| veh-amp vs. 0 | t9=3.383 | p=0.0081* |
| gal-amp vs. 0 | t9=1.348 | p=0.2106 |
| infusion x injection | F1,35=7.579 | p=0.0093* |

**Fig 5C (vehicle vs. gallein i.p., saline vs cocaine)**

| veh-coc vs. 0.0 | t6=3.671 | p=0.0104* |
| --- | --- | --- |
| gal-coc vs. 0.0 | t6=2.877 | p=0.0282* |
| veh-coc vs. gal-coc | t12=0.251 | p=0.805 |
